# 3D Stretchable Arch Ribbon Array Fabricated via Grayscale Lithography

**DOI:** 10.1038/srep28552

**Published:** 2016-06-27

**Authors:** Yu Pang, Yi Shu, Mohammad Shavezipur, Xuefeng Wang, Mohammad Ali Mohammad, Yi Yang, Haiming Zhao, Ningqin Deng, Roya Maboudian, Tian-Ling Ren

**Affiliations:** 1 Institute of Microelectronics, Tsinghua University, 100084, Beijing, China; 2 Department of Mechanical Engineering, Southern Illinois University, Edwardsville, IL 62026, USA; 3 Department of Chemical and Biomolecular Engineering, University of California, Berkeley, CA 94720, USA

## Abstract

Microstructures with flexible and stretchable properties display tremendous potential applications including integrated systems, wearable devices and bio-sensor electronics. Hence, it is essential to develop an effective method for fabricating curvilinear and flexural microstructures. Despite significant advances in 2D stretchable inorganic structures, large scale fabrication of unique 3D microstructures at a low cost remains challenging. Here, we demonstrate that the 3D microstructures can be achieved by grayscale lithography to produce a curved photoresist (PR) template, where the PR acts as sacrificial layer to form wavelike arched structures. Using plasma-enhanced chemical vapor deposition (PECVD) process at low temperature, the curved PR topography can be transferred to the silicon dioxide layer. Subsequently, plasma etching can be used to fabricate the arched stripe arrays. The wavelike silicon dioxide arch microstructure exhibits Young modulus and fracture strength of 52 GPa and 300 MPa, respectively. The model of stress distribution inside the microstructure was also established, which compares well with the experimental results. This approach of fabricating a wavelike arch structure may become a promising route to produce a variety of stretchable sensors, actuators and circuits, thus providing unique opportunities for emerging classes of robust 3D integrated systems.

Since the rise of the semiconductor industry in the 1960s, standard planar lithography processes have been used for producing transistors and integrated circuit (IC) chips, which are still widely used in current microelectronic techniques^1^,^2^,^3^,^4^. Two-dimensional patterns with vertical sidewalls are the most important feature for planar lithography. However, with the developments in microelectronic technologies, especially the advent of wearable devices and bio-sensors, microstructures with 3D morphology are critical to extend their potential practical applications including microfluidic systems^5^, artificial eyes^6^, and electronic skin^7^. Moreover, these unique microstructures may induce some interesting phenomena, like surface-enhanced Raman scattering^8^, localized surface plasmon resonance^9^ and frequency-selective surface^10^. For 3D microstructures, the most challenging procedure is the fabrication of smooth curved surfaces. So far, there are several principal approaches to fabricate 3D topographies, such as electron beam lithography^11^, nanoimprint lithography^12^, capillary force lithography^13^ and grayscale lithography^14^,^15^,^16^. Among those approaches, grayscale lithography has become the most common technique to fabricate 3D structures due to its compatibility with standard IC process, large-scale preparation for commercial industry, and controllable morphology using proper mask design. Polydimethylsiloxane (PDMS) 3D microfluidic structures^15^ and high-quality grayscale images^17^ can be achieved by using the discrete/continuous grayscale photolithography.

To date, much attention and interest have been focused on the foldable silicon microstructures which have been fabricated by different methods and exhibit great potential in flexible electronics^7^,^18^,^19^,^20^,^21^,^22^. J. He and co-workers have prepared wavelike silicon ribbons by peeling pre-strained elastomers bonded on the flat silicon ribbons, and used them to fabricate stretchable p-n diodes and metal oxide semiconductor field-effect transistors^21^. Recently, proper fractal designs for the silicon ribbon that can endure much larger plastic deformation than that of polymer materials has been demonstrated^22^. Nevertheless, most of those stretchable silicon structures are in 2D geometries. In addition, as an important common structural and dielectric material used in micro-electromechanical systems (MEMS), ICs and sensors, the 3D stretchable silicon dioxide ribbon has rarely been investigated^23^,^24^. It is hypothesized that the silicon dioxide material exhibit special mechanical properties because of this unique wavelike architecture. By thermal decomposing the polynorbornene sacrificial materials, Wu and co-workers have fabricated the silicon dioxide microchannels. The designed geometric structures display large permanent shape deformation due to the vaporization caused by the decomposition of sacrificial materials at high temperature, and resulting in curved surface^24^. However, the shape of the arch-like surfaces produced by this method is not controllable and also may result in failure of the microchannel if thin silicon dioxide layer is used to create the microchannel.

Herein, we report the fabrication of the 3D wavelike silicon dioxide ribbons via grayscale lithography and dry etching processes. We show that a proper grayscale mask design is critical to fabricate gently sloped arch structures, whose design may be guided by the results of morphology simulation. The nonplanar lithography and releasing processes are also different from the standard microfabrication and influence the final wavelike structures. Moreover, the dynamic mechanical property of the arch structure up to its fracture point is investigated in detail by atomic force microscopy (AFM) and finite element analysis (FEA). The simulated result has a good consistency with experimental result, and exhibit Young modulus and fracture strength values of 52 GPa and 300 MPa, respectively. This paper demonstrate that grayscale lithography approach can be extended to fabricate any other stretchable structures, including 3D integrated systems with potential applications in microelectronic technology.

## Mask Design

**Figure 1:**
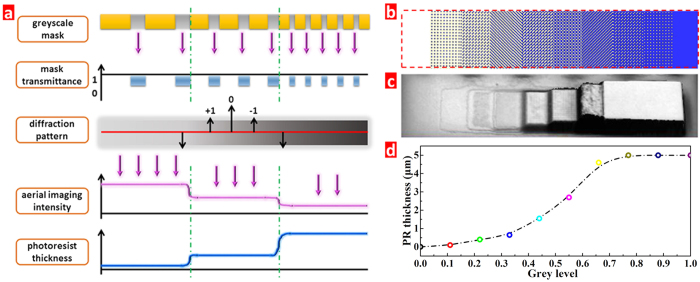
(**a**) The working principle of grayscale lithography process, (**b**) ten grey level design for calibration, (**c**) optical image of the calibration sample after developing using the ten grey level mask, and (**d**) their residual PR thickness for the normalized grey levels.

We achieved the grey-scale lithography by using pixels with different ratios of transparent-to-opaque area. The pixel number and size per area play a key role in determining the discreteness or continuity of the transferred topography^15^. Therefore, a proper mask design is critical to the grayscale lithography. To achieve different heights of the PR template along the horizontal direction, several grey levels need to be designed on the mask. Figure 1a shows the working principle of grayscale lithography. From the viewpoint of physical optics, the optical projection can be regarded as a spatial filter. First, the spatial frequency spectrum of the object from the mask can be obtained by the Fourier transform, and then passed through an optical demagnification system with a ratio of 1:5 to the diffraction surface. During the transmission process of the spatial frequency spectrum, some spectral orders of the object information are lost due to the limited numerical aperture. Finally, Fourier inversion is used to transform the diffraction image to the PR layer. For the grayscale lithography, the maximum linewidth of the mask is decided by the resolution of the stepper itself. On one hand, if the pixel size in the mask is larger than the minimum resolution, all spectral orders can pass through the system to the wafer plane and the detailed information of the object image can be perfectly projected on the PR layer, see Figure S1. The smaller the object size, the more spectral information is lost. On the other hand, when the object size is smaller than the minimum resolution, only the zero-order spectral information is left, which provides almost homogeneous light intensity on the PR layer^25^. Therefore, one can obtain a curvilinear microstructure caused by the gradient light intensity distribution.

As mentioned above, the fabrication of the curved surface can be achieved by proper mask layout with gradient light transmittance. Because the minimum resolution of our stepper is 2.5 μm, a 3 × 3 matrix unit with each pixel size of 1 μm has been designed for the calibration (Figure S2). Figure 1b shows a ten-grey level mask with transmittance value from 100% to 0%, which has a gradient value of 11%. For the ultraviolet exposure, high and low exposure doses cause overexposure and underexposure, leading to entire removal of PR or PR residues, respectively (Figure S3). Under the moderate exposure dose of 260 mJ/cm^2^, Fig. 1c shows that at-best, an eight-order grey level is obtained rather than ten-order. This is due to a nonlinear response characteristic between the residual PR thickness and the light intensity for grey levels. It is noted that the normalized grey levels in Fig. 1d exhibit a large height difference between the 0.33 and 0.66 levels, indicating that more grey levels are needed to get the smooth curved surface.

**Figure 2:**
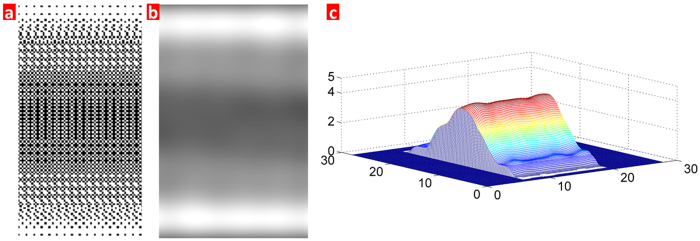
(**a**) The grey levels design for the arch structure, (**b**) the simulation result of grey distribution corresponding to the mask in panel (a), and (**c**) the 3D morphology of the simulation result after development.

To choose an optimized mask design for grayscale lithography, Matlab software is used to simulate the arch morphology after developing, and the main simulation process is shown in Figure S4. In this simulation, we increase the pixel matrix unit up to 4 × 4 which has a lower gradient light transmittance value of 6.3%, as shown in the pixel units design in Figure S5. Figure 2a shows the mask design for the arch structure, and the light transmittance has a gradual decrease from the horizontal center axis towards its both sides. From the simulation result in Fig. 2b, the grey gradient light distribution can be seen in the diffraction plane. Based on the previous experimental results of PR thickness response to light intensity, the final morphology of the residual PR after developing is shown in Fig. 2c, where much smoother arch surface can be obtained using the seventeen grey levels. It is noted that a perfect arch structure would be achieved by less gradient light transmittance if the stepper has a smaller minimum resolution.

### Fabrication Process

**Figure 3:**
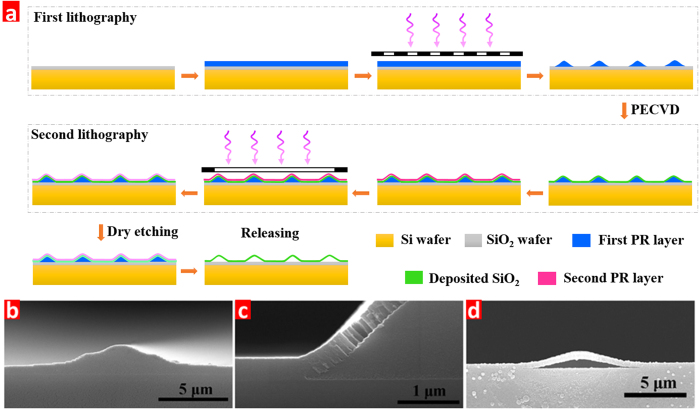
(**a**) The main fabrication processes for the 3D arch ribbon array microstructure, and cross-section SEM images after (**b**) the first lithography, (**c**) the silicon dioxide deposition (**c**), and (**d**) releasing step.

The fabrication process is illustrated in Fig. 3a, and mainly contains five steps: the first lithography, silicon dioxide deposition, the second lithography, dry etching and removal of the PR sacrificial layer. For the first lithography, a PR layer with a thickness of about 5 μm was spun on a cleaned wafer and was exposed under optimal exposure dose using the mask with seventeen grey levels, followed by the final developing. Due to the gradient distribution of the exposure dose at the different areas, the wavelike PR template can be produced. Figure 3b shows the scanning electron microscope (SEM) image of the developed sample, and peak height of the PR layer and span length are about 1.8 and 10 μm, respectively. It can be seen that the gentle slope PR template for the silicon dioxide deposition can be produced via grayscale lithography.

Before the silicon dioxide deposition, the oxygen plasma was utilized to clean the residual PR on the valley area for better adhesion between the wafer surface and the deposited silicon dioxide. Then, the silicon dioxide layer was deposited on the previous curved PR template by PECVD at low temperature. This is because the PR is composed of organic compounds and its exposure to high temperatures may damage the PR template. The SEM image in Fig. 3c shows that the silicon dioxide has been well coated on the curved PR layer, and the deposited thickness is about 400 nm.

**Figure 4:**
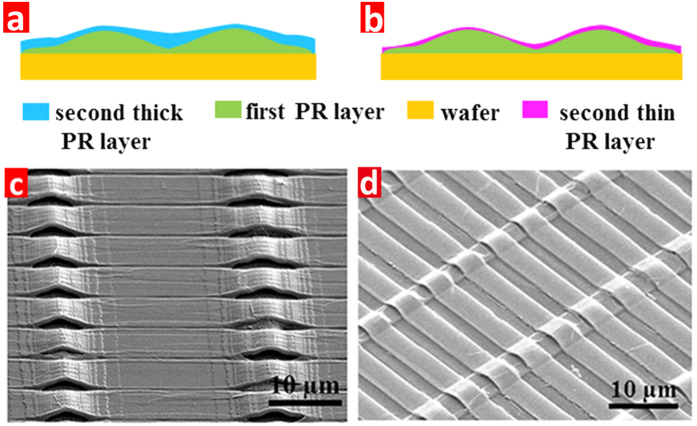
The thickness distribution illustrations for (**a**) thick, and (**b**) thin PR layers, and their SEM images after developing using the (**c**) thick, and (**d**) thin PR layers.

To form the wavelike ribbon array sequentially, a second lithography step is done using the stripe shape mask perpendicular to the previous arch structure. Because of its wavelike silicon dioxide surface, it is a challenge to achieve homogenous PR coating for the uniform exposure dose over the whole area. In general, for the thick PR, the thickness close to the arch roof is much smaller than that in the valley area, as shown in Fig. 4a. Thus it is very hard to control the exposure dose for the inhomogeneous PR layer. Figure 4c shows the SEM image of the etched result using thick PR. It can be seen that, on one hand, the overexposed area at the arch roof broadens the gap between the adjacent ridges and the arch width becomes narrow after etching. On the other hand, the underexposed area in the valley leaves residual PR, and a gap can not form between the arches after etching. Due to the usage of thick PR, homogeneous PR thickness can not be formed for the second lithography. Therefore, a thin PR is used with a thickness of 1.5 μm by spinning on the flat substrate at 2500 rpm. Although the thickness is much less than the arch altitude, the PR layer forms good conformal coating with the curved substrate, as shown in Fig. 4b, because for the thin PR, the van der Waals force between the PR solution and the substrate mainly contributes to adhesion, rather than gravity. In addition, the rough surface caused by grayscale lithography is also beneficial for the physical absorption of PR. Thus, the height difference error is much less than that of thick PR. As shown in Fig. 4d, the SEM results indicate that the arch ribbon structure can be achieved well using the thin PR for the second lithography step.

After developing, the dry etching process was carried out by using the high density plasma etching system to etch the exposed silicon dioxide layer, as shown in Fig. 3a. Figure 3d shows the SEM image of silicon dioxide arches with height of ~2 μm.

**Figure 5:**
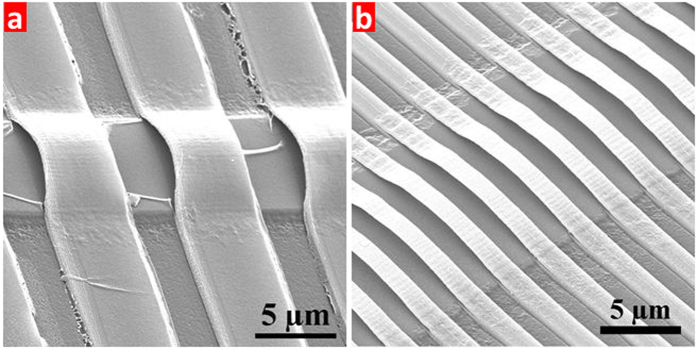
The SEM images of releasing results after fuming nitric acid treatment for (**a**) 10 min, and (**b**) 30 min.

In general, the final release process of the arch ribbon array can be directly achieved by removing the PR sacrificial layer using acetone. In fact, after the first lithography, the acetone solution can certainly lift off the PR even in pipe-like structures (Figure S6). However, the PR can not be removed by acetone solution after the second lithography and etching processes as the PR has denatured. Since there is no metal material used in this structure, we have used the fuming nitric acid to remove the denatured PR. As shown in Fig. 5a, the arch ribbon structures can be released well after 10 minutes treatment while some accumulated PR still remains on the arch surface and substrate. Although the extended immersion in acid is beneficial for the removal of PR completely, the SEM image in Fig. 5b shows that the shrink and wrinkle effect on the surface occurs on the thin arches after 30 minutes treatment. However, the oxygen plasma can be used to completely remove the residual PR (Figure S7).

## Discussion

**Figure 6:**
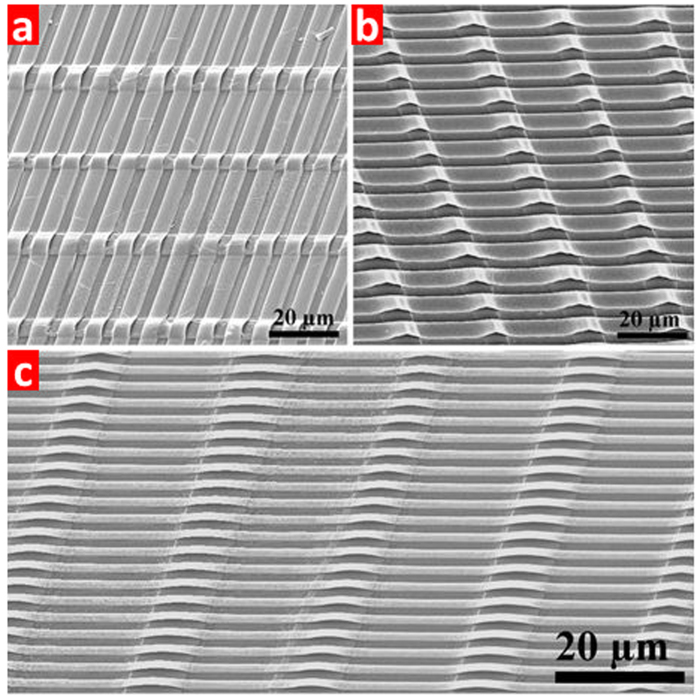
SEM images of the arch ribbon arrays with different sizes: (**a**) 6 μm width and 3 μm gap; (**b**) 4 μm width and 2 μm gap; and (**c**) 2 μm width and 1 μm gap.

Figure 6 displays the successful fabrication of different arch arrays with 6 μm, 4 μm and 2 μm arch widths. All the SEM images show that there are no defects, like crack, exfoliation or severe distortion on the arch ribbon surface, indicating that the grayscale lithography technique is an effective way to achieve high-quality 3D arch ribbon arrays.

**Figure 7:**
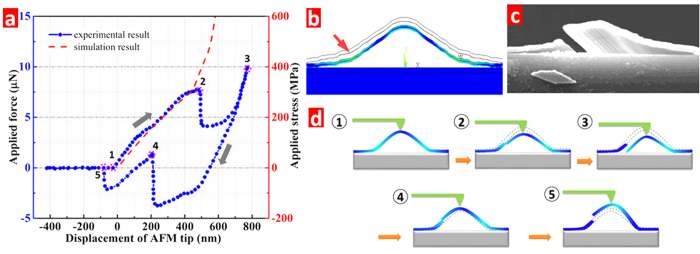
(**a**) The displacement versus applied force curves of the experimental result using the AFM tip, and simulation result using FEA method for the arch microstructure, (**b**) stress distribution inside the arch structure and the red arrow shows the maximum stress point, SEM image with incline viewing angle after the loading and unloading cycle (**c**), and (**d**) illustrations of the arch structure corresponding to those loading and unloading points in panel (a).

To investigate the mechanical characteristics of the 3D arch structure, the strain-displacement curves have been measured by AFM and also simulated by FEA. Based on a Poisson ratio of *υ* = 0.17 in the literature^26^, the simulation model of silicon dioxide arch has been established. Figure 7a shows the experimental and simulation results for the relationship between the force applied by AFM tip and the out-of-plane displacement of the arch top point up to the its fracture. The location of maximum stress and a fractured arch are shown in Fig. 7b,c, respectively, and loading/unloading characteristics of the arch is illustrated in Fig. 7d. The displacement of the AFM tip exhibits a nearly linear response to the applied force between 0–7.5 μN, which has a good consistency with the simulation result. At the displacement position around 490 nm, the applied force has a rapid decrease from 7.5 μN to 4.1 μN, indicating that fracture occurs in the arch structure. It is noted that the applied force value is reduced by almost one-half, and each segment absorbs half of the applied force for the symmetrically broken structures. The Young modulus obtained by matching the measured and simulated force-displacement curves is 52 GPa, which is smaller than that of silicon dioxide with 500 nm thickness reported by Gianola^27^. Moreover, the fracture strengths of simulated and experimental results have a similar value of about 300 MPa, which is close to the previous reports^27^,^28^. Finite element simulation was used to determine the fracture stress and the location of fracture, and the results are shown in Fig. 7b. It is shown that the maximum stress point is not on the peak on which the force has acted, but about its one-third height position, which is also demonstrated by the SEM result (Fig. 7c). The strain-displacement curve of the experimental result shows a non-linear variation at the displacement range of 500–700 nm due to relaxation effect after cracking, however, the force-deformation relation was not simulated after fracture.

Interestingly, when unloading the force in the AFM tip, there is a negative force at the displacement position less than 550 nm. The negative force means that the tip experiences the force from the arch structure, which has an ability to recover to its natural state. The reason for the sharply increased applied force around 200 nm is the contact interaction between the right and left branches of the cracked arch. As the intact arch shares the force later, the slope of applied force versus displacement becomes the same as the slope before its crack. It is noted that the final position of the tip has not overlapped with its starting point after the applied force is totally released from arch structure, and displays about 80 nm higher arch than its original displacement. This suggests that the arch structures already contain internal stresses after fabrication, which is mainly induced by the fuming nitric acid releasing treatment^29^. Moreover, the arch structure can undertake the thermal shock at 350 °C (Figure S8). The results show that 3D arch ribbon microstructure with outstanding mechanical and thermostability properties can be utilized in flexible devices and in a hostile environment.

## Conclusion

By combination of the grayscale lithography, PECVD and etching processes, we have successfully fabricated 3-D architecture with wavelike ribbon array. Several processes including the grey mask design, non-planar lithography and lift off have great influence on the final morphology. The established theoretical model for the morphology after first lithography agrees well with the experimental results, which can guide the mask design. Using the optimal process parameters, the silicon dioxide arches with widths of 2, 4 and 6 μm were fabricated. Moreover, the mechanical properties of the arch ribbon microstructure were discussed in detail using experimental and simulation results. Due to the unique 3D arch structure, the silicon dioxide arch displays smaller Young modulus. The results indicate that this fabrication route is very promising in realizing other 3-D microstructures using proper mask design, which has great potential applications in the domain of flexible and stretchable sensors and devices.

## Methods

### Sample fabrication

A 4 inch Si wafer with silicon dioxide thickness of 3000 Å was used for the arch structure fabrication. For the first lithography, positive-tone AZ4620 PR layer was spun on the wafer at 5000 rpm, and then prebaked at 95 °C for 90 s. The lithography was carried out by the stepper ASML PAS 5000/55, which mainly contains an I-line light source (365 nm), a high precision optical projection system and a mechanical control system. After developing, post-baking was carried out to solidify the PR arch, and O_2_ plasma treatment was used to clean the residual PR on the valley using Drytek Megastrip 6. The silicon dioxide was deposited on the wafer by PECVD process using CAS-PECVD-2 at a low temperature of 100 °C. The SiH_4_ and N_2_O gases were utilized to set off a chemical reaction forming silicon dioxide layer at a flow rate of 10 sccm and 40 sccm, respectively. The chamber pressure was kept at 21 Pa and the radio frequency power was fixed at 50 Watts. For the second lithography, thick AZ4620 PR has been spun on the previous sample using the process parameters above, while another thin positive AZ601 PR layer was spun at 2500 rpm and prebaked at 100 °C for 90 s for the comparison with thick PR. Then, a relatively fast exposure with an expose power of 4.3 mW/cm^2^ for 22 s was conducted using a Cannon PLA 550 mask aligner. After developing using acetone or fuming nitric acid, the post-baking has been carried out at 115 °C, which is beneficial for the etching process. The etching has been conducted to get a wavelike arch array by the high density plasma etching system using ULVAC NE550 for 90 s.

### Characterization

The surface morphology of the prepared samples was carried using a Quanta FEG 450 SEM. To test the mechanical property of the micro-ribbon structure, the applied force has been acted on the arch by the AFM tip.

## Additional Information

**How to cite this article**: Pang, Y. *et al*. 3D Stretchable Arch Ribbon Array Fabricated via Grayscale Lithography. *Sci. Rep.* **6**, 28552; doi: 10.1038/srep28552 (2016).

## Supplementary Material

Supplementary Information
