# Supplementary material for: 3D Stretchable Arch Ribbon Array Fabricated via Grayscale Lithography
Source: Sci Rep. 2016 Jun 27;6:28552. doi: 10.1038/srep28552 (PMC4921820; doi:10.1038/srep28552)
Supplement: Supplementary Information [file srep28552-s1.pdf]

3D Stretchable Arch Ribbon Array Fabricated via Grayscale Lithography

Yu Pang[1+], Yi Shu[1+], Mohammad Shavezipur[2,3+], Xuefeng Wang[1], Mohammad Ali Mohammad[1], Yi Yang[1], Haiming Zhao[1],Ningqin Deng[1], Roya Maboudian[3], and Tian-Ling Ren[1]*

[1]Institute of Microelectronics, Tsinghua University, 100084, Beijing, China

[2]Department of Mechanical Engineering, Southern Illinois University, Edwardsville, IL 62026, USA

[3]Department of Chemical and Biomolecular Engineering, University of California, Berkeley, CA 94720, USA

[*]corresponding author: RenTL@tsinghua.edu.cn

[+]These authors contribute equally to this work

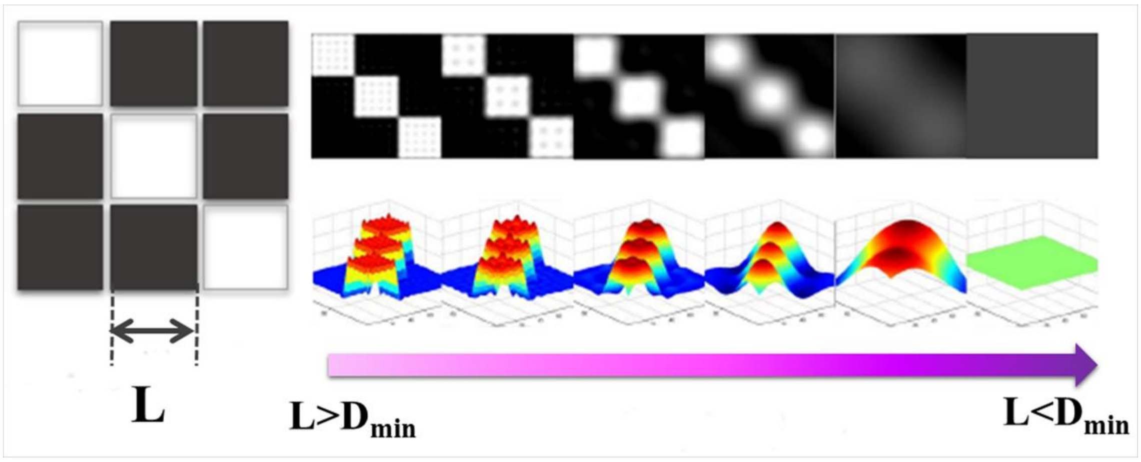

Figure S1. A 3×3 matrix unit with nine square pixels, and optical images of the matrix unit shows that the object information has been gradually lost with deceasing L size. The simulation results corresponding to each condition have also been displayed.

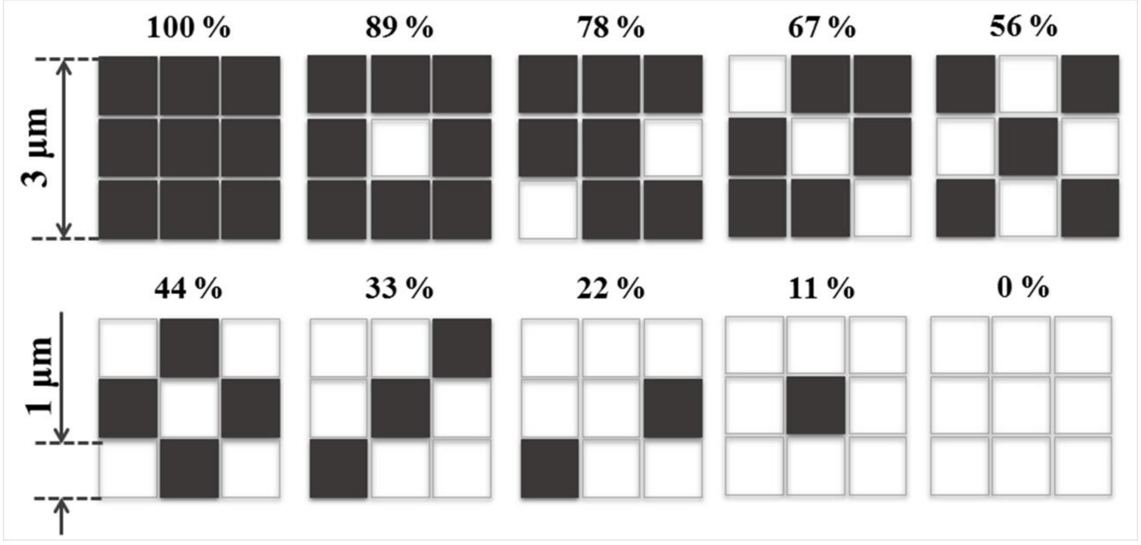

Figure S2. Ten grey levels design: a 3×3 matrix unit with a gradient transmittance value of 11 %.

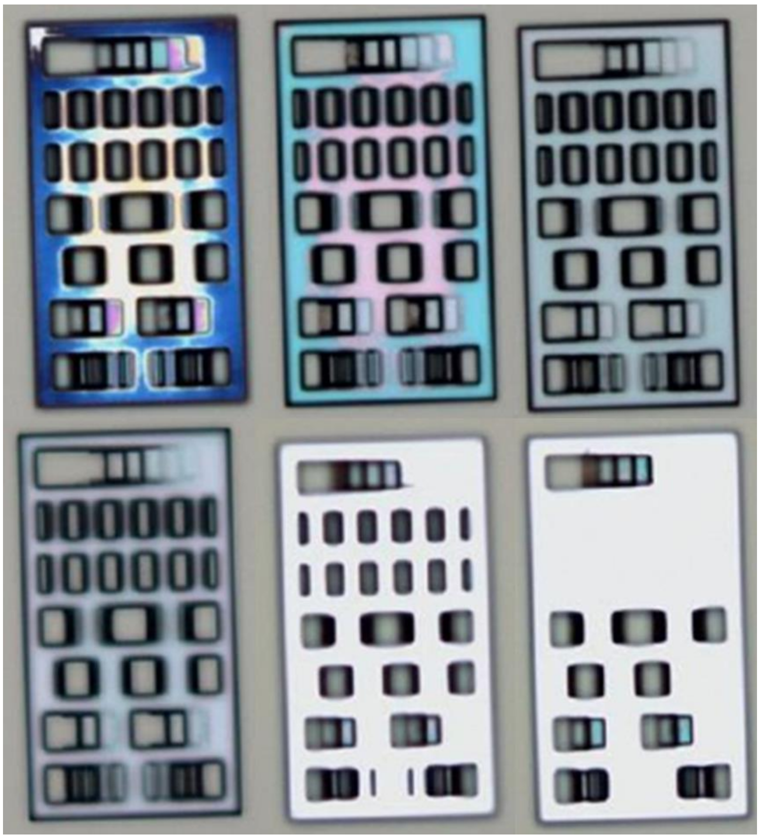

Figure S3. Optical image after developing at different exposure dose of 220, 240, 260, 280, 300, and 320 mJ/cm^2 from upper right to lower left.

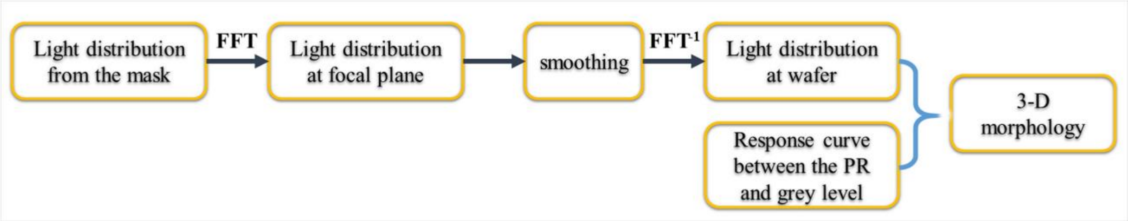

Figure S4. The main process of simulated morphology after developing.

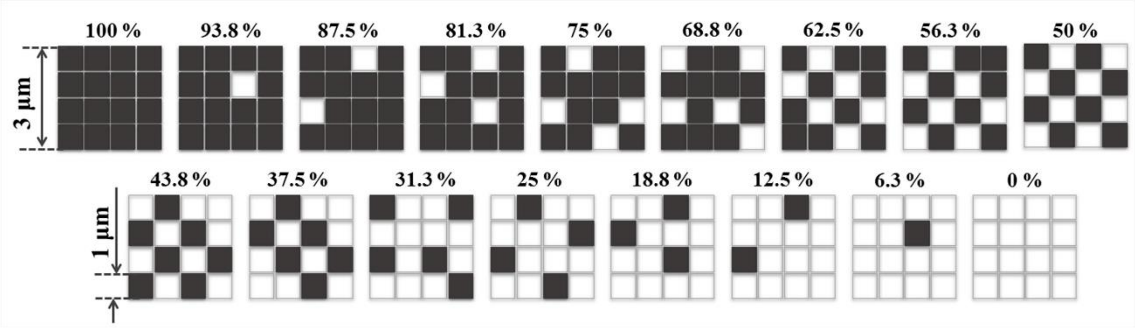

Figure S5. The seventeen grey levels design: a 4×4 matrix unit with a gradient transmittance value of about 6.3 %.

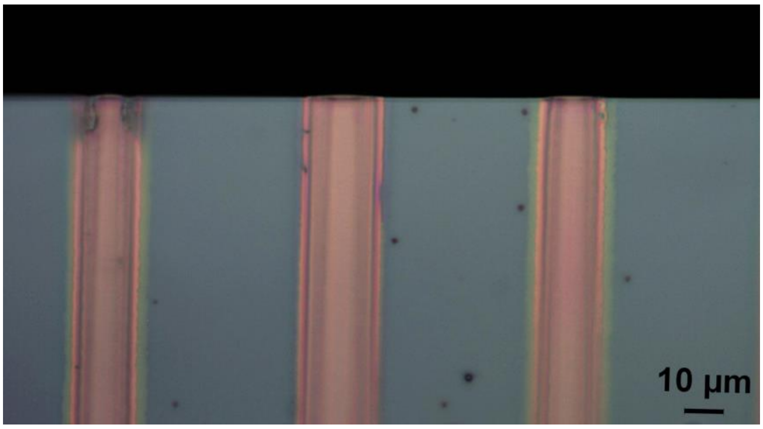

Figure S6. The optical micrograph of the pipe-like silicon dioxide developed after the first lithography.

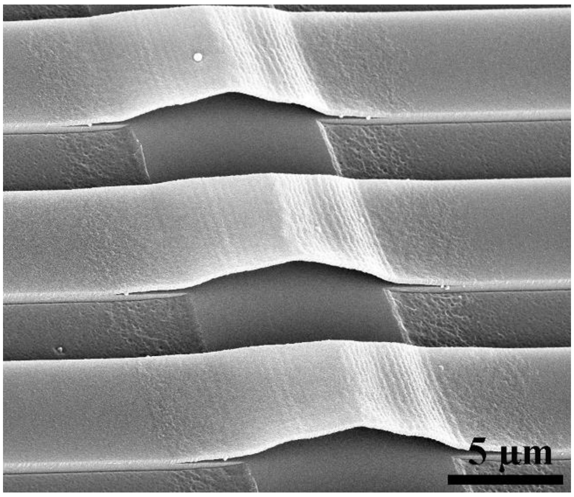

Figure S7. The SEM image of arch ribbon structure after the 30 min treatment of oxygen plasma.

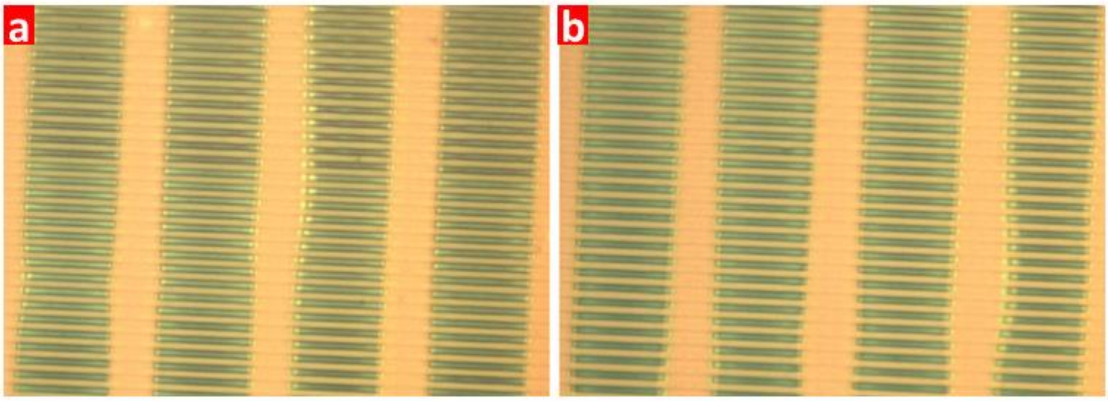

Figure S8. The optical images of the arch ribbon array (a) before, and (b) after heat treatment.
